# Employing Hybrid Image Processing for Grape Sorting

**DOI:** 10.3390/s26206574

**Published:** 2026-10-16

**Authors:** Simon Wezstein, Michael Stelzl, Michael Heizmann

**Affiliations:** 1 Institute of Industrial Information Technology, Karlsruhe Institute of Technology, 76131 Karlsruhe, Germany; michael.heizmann@kit.edu; 2 MSTVision GmbH, 65462 Ginsheim-Gustavsburg, Germany; michael.stelzl@mstvision.de

**Keywords:** grape processing, system design, real-time processing, FPGA-based sorting, Linux

## Abstract

This contribution presents a practical realization of hybrid image processing for grape sorting. Hybrid image processing combines FPGA (Field Programmable Gate Array), GPU (Graphics Processing Unit), and CPU (Central Processing Unit) hardware with both rule-based and CNN (Convolutional Neural Network)-based algorithms to satisfy real-time constraints at high data throughput. A detailed end-to-end latency analysis of the complete sorting pipeline is provided. Our initial solution achieves a mean latency of 10.7 ms with a 99.9th percentile of 13.2 ms and a maximum latency of 14.0 ms. Our investigation reveals that the primary bottleneck lies in data handling rather than CNN inference. Furthermore, FPGA-based and CPU-based preprocessing approaches are compared using identical image data. A CPU-/GPU-based approach achieves a mean latency of 7.3 ms with a 99.9th percentile of 13.0 ms and a maximum latency of 13.5 ms. Finally, an improved FPGA architecture is proposed and evaluated, demonstrating improved timing performance. The improved FPGA solution achieves a mean latency of 9.0 ms with a 99.9% percentile of 10.6 ms and a maximum latency of 11.3 ms. The machine’s capability is evaluated at a grape processing throughput of 5.6 t/h.

## 1. Introduction

Many (optical) sensor-based sorting applications require balancing conflicting objectives: Image analysis complexity, processing latency, material throughput, and optical resolution. Our concept, “hybrid image processing”, addresses this challenge by combining rule-based and CNN-based algorithms with heterogeneous processing hardware, including FPGAs, GPUs, and CPUs.

This article is a revised and expanded version of a paper entitled “Employing hybrid image processing for grape sorting” presented at the 11th Sensor-Based Sorting & Control Conference (SBSC 2026), Aachen, Germany, 17–18 March 2026 [1].

In this contribution, we show how the hybrid image processing architecture concept, whose timing we analyzed in [2], can be transferred to a practical industrial application. While built on a theoretical foundation, there has been no published practical implementation since. The practical implementation is shown in the field of grape processing for winegrowers. The field of grape processing is a cost-sensitive yet challenging field. This challenge was addressed in collaboration with Scharfenberger GmbH & Co. KG (Bad Dürkheim, Germany) and Strelen Control Systems GmbH (Büttelborn, Germany). Wine growers are usually not experts in sorting machines and machine vision, and furthermore, such a solution is only used for a short time during the season. The visual appearance of grapes varies from season to season. A sensor-based sorting process in this area must balance material throughput, classification performance, purchase price, and ease of use. Furthermore, a machine of this type is purchased to be used for up to 20 years. Our approach takes this into account by relying on standard hardware and interchangeable classifiers. Our refined hybrid image processing approach uses an RGB line scan camera with a Basler frame grabber with user-customizable FPGA logic for image acquisition, object detection, and tracking. The classification is performed by CNNs, inferred on a GPU. We provide a detailed overview of the image processing pipeline with its latencies and throughput.

There are numerous grape sorting machines on the market, for example: ARMBRUSTER Kelterei-Technologie GmbH (Güglingen-Frauenzimmern, Germany) [3] offers a machine first marketed in 2016 focusing on the removal of non-grape material from the product stream. The machine was developed in collaboration with Fraunhofer IOSB (Karlsruhe, Germany), Hochschule Geisenheim (Geisenheim, Germany) and Ingenieurbüro Waidelich (Kirchentellinsfurt, Germany) [4]. It uses cameras in the visible and near-infrared spectrum. According to a reseller, WiLLMES GmbH (Lorsch, Germany) [5], the camera runs at up to 18 kHz and uses 128 pneumatic rejection nozzles with a capacity of 8 t/h.

Pellenc America, Inc.’s (Santa Rosa, CA, USA) [6] sorter is specified to process 2000 berries per second and up to 12 t/h. Palmaz Vineyards (Napa, CA, USA) [7] reports the use of 3D image processing with a Pellenc machine. According to the authors, sorting accuracies close to 100% can be achieved.

Sensor-based sorting has gained in popularity in recent years. However, a gap remains between laboratory-scale research and industrial applications [8]. According to Kroell et al. [8], future research will benefit from the use of deep learning methods. Tang et al. [9] use heterogeneous processing hardware (1 FPGA, 1 PC and 1 GPU) and CNN-based algorithms to sort cells. They reach end-to-end latencies of less than 3 ms while processing 200 events per second. The process involves the inference of a customized U-Net architecture via PyTorch 1.10.2 on a Windows PC. Wang et al. [10] use an FPGA to implement a complete cell sorting system including a classification CNN. They achieve a classification latency of 34 μs and reach a throughput of 29,200 objects per second.

Existing systems demonstrate the feasibility of optical grape sorting. However, publicly available information on the employed processing architectures is limited.

The questions addressed in this work are:

1. Is the hybrid image processing architecture suitable for a practical grape sorting application?
2. What end-to-end latencies can be achieved in practice?
3. To what extent can FPGA-based preprocessing be replaced by host-side CPU processing?
4. Can the system latency be further reduced through an improved FPGA architecture?

## 2. Materials and Methods

This section covers the concept of the machine and our evaluation method.

### 2.1. Hybrid Image Processing Approach

The developed concept of “hybrid image processing” seeks to combine standard machine vision algorithms with recent developments in neural networks, especially convolutional neural networks (CNNs). In [2], we showed a novel concept to use an FPGA to reduce the data throughput for a low-throughput classifier running on a Graphics Processing Unit (GPU).

**Figure 1:**
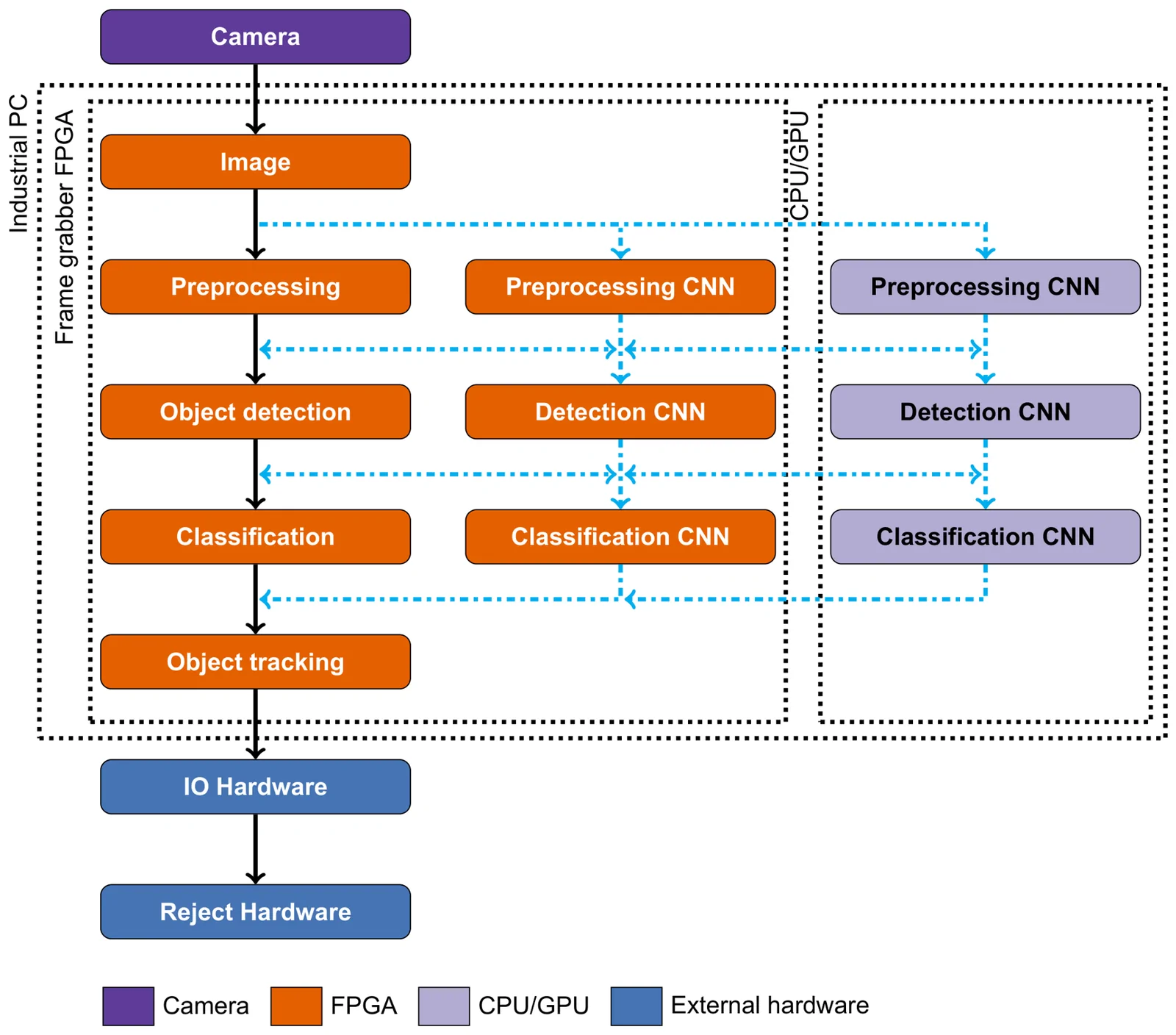
Approach design overview. On the left side, the image processing pipeline is shown with rule-based algorithms. Preprocessing, object detection, and classification may be changed to a CNN counterpart, running either on FPGA or CPU/GPU. The usage of multitask neural networks is not illustrated, but is possible, too. The arrows indicate the data flow, whereas the dotted blue arrows denote CNN processing data paths. Modified version from [12].

Fundamentally, the control and processing scheme of a sorting system is structured as shown in Figure 1. On the hardware side, there is an industrial PC with an AMD64 CPU and a Basler frame grabber. At first, the system used the frame grabber’s FPGA for the whole process. On the algorithm side, all processing steps were built using rule-based implementations. We analyzed its timings in [11]. The new architecture is built on this existing system. Every relevant processing step (the right columns in Figure 1) may be interchanged with neural network counterparts. The term “hybrid” stands for two dimensions of hybridization: on the hardware side, determining if an algorithm should be implemented into the FPGA design or run on the host, with an additional NVIDIA GPU. In addition, on the software/algorithm side, it determines whether the rule-based or neural network approach should be used. The hybrid image processing concept originated from a ZIM-funded research project conducted in 2021. Due to PC-to-FPGA upload bandwidth limits and FPGA resource limits, some possible processing pipelines are impractical, such as using a preprocessing CNN, inferred on the GPU, since a possible high-bandwidth data stream would need to be transferred back to the FPGA with low latency. While FPGA-to-host transfers utilize DMA (Direct Memory Access), host-to-FPGA transfers rely on MMIO (Memory-Mapped I/O) register accesses. Consequently, only small amounts of information, such as object identifiers and classification results, can be returned to the FPGA efficiently.

**Figure 2:**
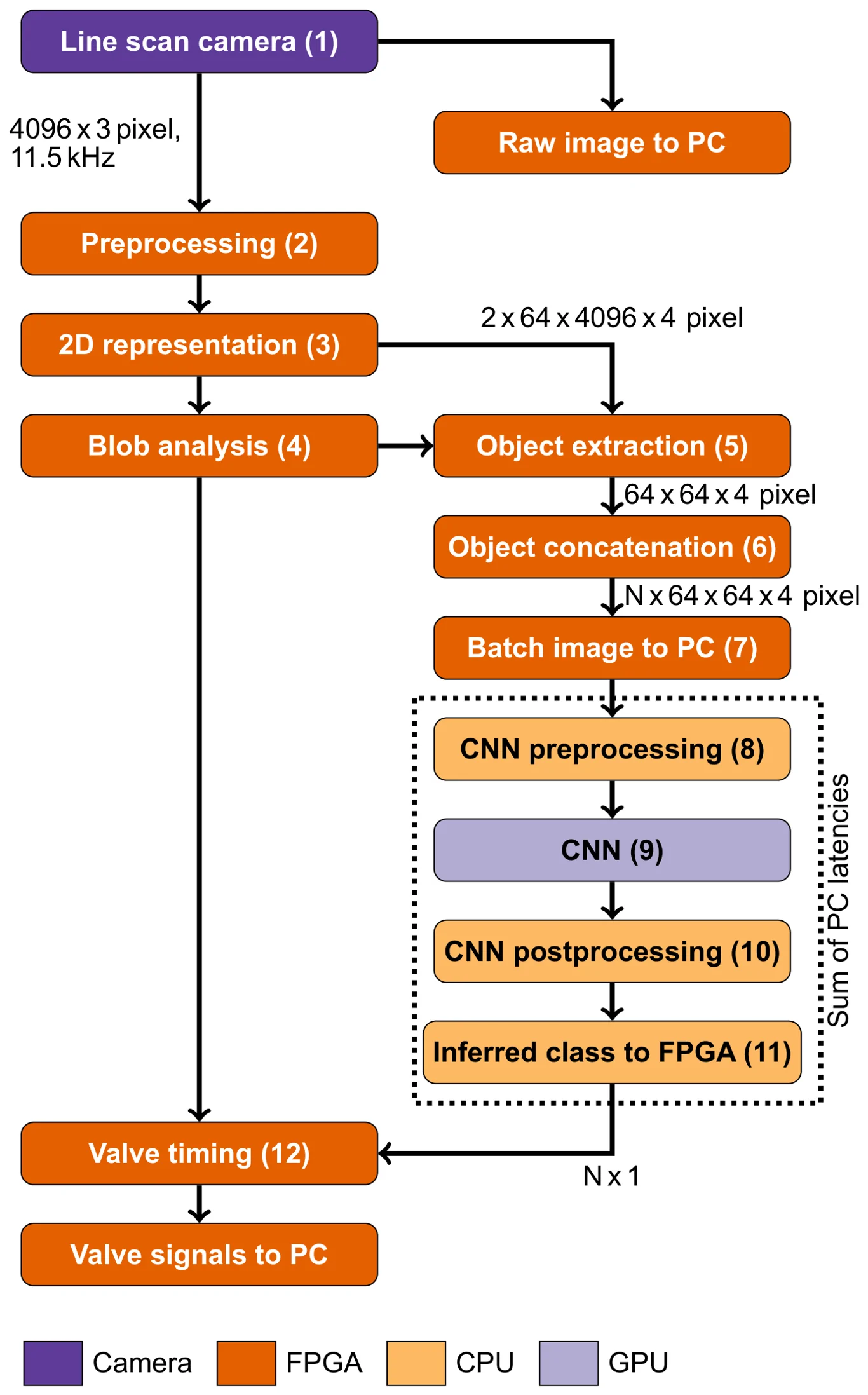
System data flow model, all shown image dimensions are noted without our extra timing information, needed for measurements. The arrows indicate the data flow.

One of the practically usable combinations of our research is shown in Figure 2. It represents a workflow, using the FPGA for all tasks except the object classification step. This combination provides good real-time capabilities due to the precise synchronization of line acquisition and object detection enabled by high-resolution, high-frequency timers. On the other hand, state-of-the-art classification CNNs can be employed, provided their latency matches the speed of the process. While the CNN inference itself is executed on the host GPU and therefore introduces non-deterministic processing latency, the object timing information remains anchored to the FPGA domain. Consequently, as long as the classification result is returned before the latest admissible decision point, the final reject actuation retains the deterministic timing accuracy of the FPGA-based tracking system. This is the combination which we used for the real-world application of grape sorting.

### 2.2. Real-World Application

Scharfenberger currently offers an optical sorter, which only uses color information for processing. Higher-level features like shape or texture are not considered. To update this machine, some hardware conditions are set:

- Distance between a line scan camera and the reject nozzles ($l_{rej}$) approx. 60 mm.
- Belt speed ($v_{b}$) approx. 3 m/s.
- Belt and inspection width approx. 800 mm.
- Pixel resolution $l_{px}\;\approx$ 0.25 mm/px.
- 8-Bit RGB Camera.
- 4096 px per line.

The object diameter is expected to be around 10 mm. This results in an object bounding box height/width of approx. 40 px. To use a power of two, the size for an object patch ($s_{p}$) was defined to be 64 px.

The system’s line rate ($f_{L}$) is calculated by:

(1) $$f_{L}=\frac{v_{b}}{l_{px}}=\frac{3\;m\;s^{-1}}{0.25\;\mathrm{mm}}=12\;\mathrm{kHz}$$

The theoretical line rate of 12 kHz corresponds to approximately 147 MB/s of 8-bit RGB pixel data. At full capacity, a product throughput of 10 t/h is expected. Assuming an average berry weight of approximately 1 g, which is consistent with the range of 0.83 g to 3.18 g reported by [13] for different grape varieties, this corresponds to roughly 10 million berries per hour, or about 2800 berries per second. Due to variations in the actual pixel resolution of the deployed machine, which result from the realized mechanical and optical geometry and parameters, the target line rate is adjusted to maintain approximately square pixels.

The theoretical maximum allowed system latency ($t_{max}$) is calculated by:

(2) $$t_{max}=\frac{l_{rej}}{v_{b}}=\frac{60\;\mathrm{mm}}{3\;m\;s^{-1}}=20\;\mathrm{ms}$$

This value includes the line accumulation latency of an object.

The theoretical maximum allowed system latency, with respect to the maximum object height ($t_{max,obj}$), is calculated by:

(3) $$t_{max,obj}=t_{max}-s_{p}\cdot f_{L}^{-1}=20\;\mathrm{ms}-64\;\mathrm{px}\cdot {\left(12\;\mathrm{kHz}\right)}^{-1}=14.7\;\mathrm{ms}$$

The practical maximum latency is lower due to the response time of the pneumatic reject system.

To allow the end customer to tune the machine to his product, the concept “Bring your own AI” is employed, meaning the software is built so that the classifier may be changed by the user. Therefore, the portable data format “ONNX” [14] is used. Strelen’s software enables the user to train new classification models from data captured by the machine. Training can be done on the machine itself. Because of this, this contribution does not focus on the CNN model, but on the application of the proposed architecture.

**Figure 3:**
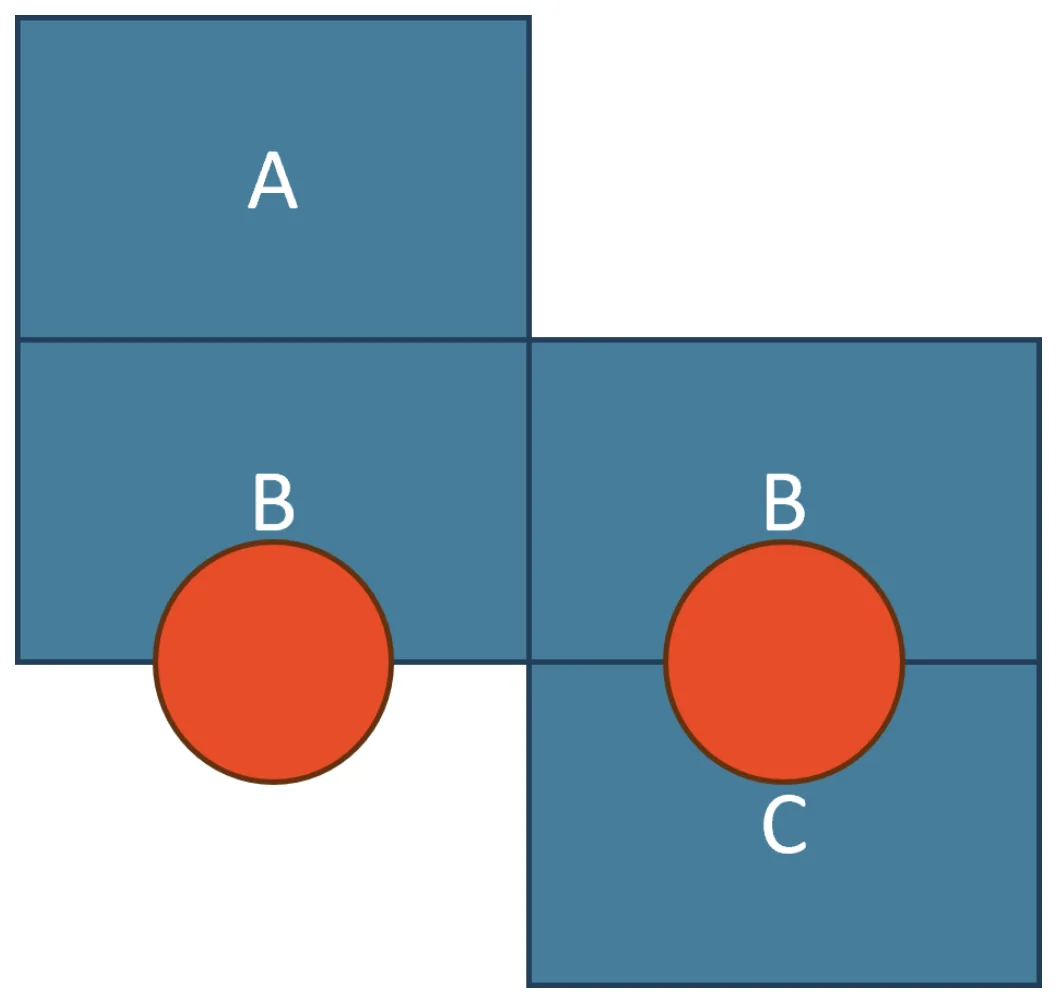
Data layout of the 2D context for blob analysis. A block of lines (labeled A, B, and C) is always paired with the previous block of lines (B with A, then C with B). This ensures that objects overlapping a context boundary, illustrated by the orange circle, are processed once all required lines are available.

**Figure 4:**
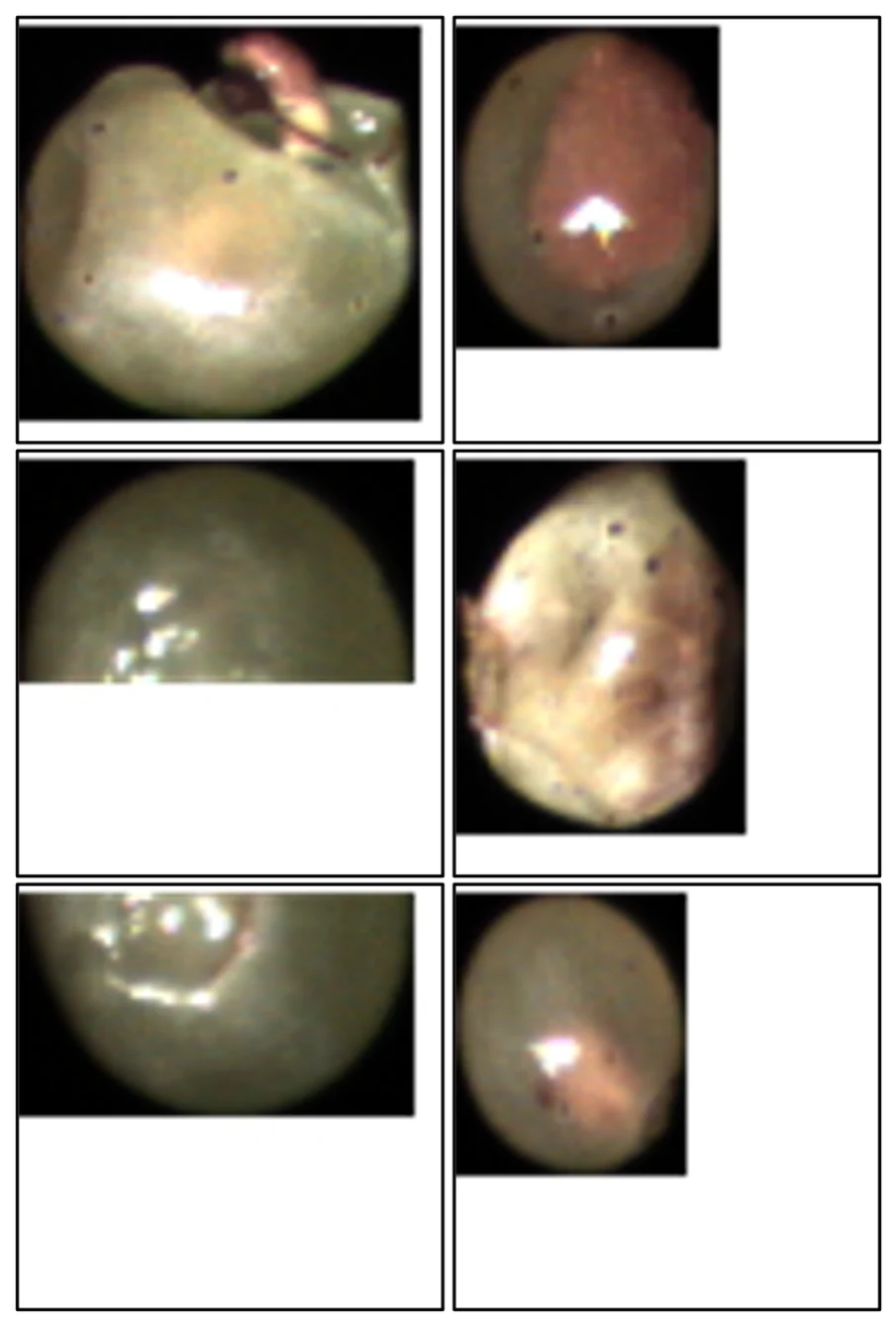
A set of extracted objects. The lower two images on the left show an object, which was cut in half due to the height exceeding the limits.

Figure 2 shows the data flow diagram of the system: The data stream of the line scan camera (1) is preprocessed (2) and then packed into a 2D representation (3). The preprocessing comprises geometrical correction and segmentation. The 2D representation always combines two blocks of 64 lines, which is the target size for the classification CNN (9). To prevent objects from being cut in half, the most recent 64-line block is repeated and concatenated to the now most recent 64-line block. This doubles the data rate to be processed by the blob analysis (4), which takes place afterward. Another downside is the possible latency increase. This concept is visualized in Figure 3. If an object is higher or wider than 64 pixels, it is divided into multiple sub-blobs. The detected objects are tracked (12) for rejection, if necessary. In parallel, the current 2 by 64 lines data block (double block) is stored in RAM to extract the detected objects (5); see Figure 4. All objects found in one double block are concatenated to one object batch (6) and transferred to the PC (7). A maximum batch size of 32 is allowed; otherwise, the accumulated data are split into multiple batches. After processing the CNN classifier (8–10) with the CPU and GPU on the host system and transferring the class prediction back to the FPGA (11), the decision is made to keep or reject the object. If the classifier is too slow, the FPGA’s configurable default action (reject or keep) applies.

### 2.3. Assessment Approach

Our first experiment is the evaluation of the used CNNs with our hardware to determine how long the plain inference will take for specific batch sizes.

The second experiment is a simulation of the real machine. We create an FPGA design with a synthetic image source. Instead of a line scan camera, we replay an image, captured in field tests, infinitely. Timestamps are inserted at every relevant pipeline step.

To determine if the image processing steps shown in Figure 2 require FPGA acceleration for our application, we port them to the PC. The data flow is organized as follows:

1. Line acquisition,
2. Append FPGA timestamps,
3. Transfer to PC in batches of 16 lines,
4. Add to larger buffer,
5. Perform preprocessing (HSI thresholding, Morphological operations),
6. Do a blob analysis,
7. Extract and concatenate blobs,
8. CNN pipeline,
9. Transfer information to FPGA,
10. Add FPGA timestamps,
11. Relay data and timestamps to PC.

The data are then evaluated afterwards. Processing 16 lines at 11.5 kHz is equivalent to 719 Hz or 1.4 ms at the PC-pipeline’s input. We use an internal ring buffer of 9 times 16 lines for raw and binary data. The buffer was allocated slightly larger for better debugging and less frequent buffer wraparounds. Every time a new input arrives, a blob analysis is performed and merged with the previously observed data. If an object ends or exceeds the 64-line maximum height, the data are extracted from the raw input, concatenated with other samples, and fed to the CNN pipeline. The theoretical worst-case latency between the first object line and object extraction is $80\;\mathrm{lines}\cdot {\left(11.5\;\mathrm{kHz}\right)}^{-1}=7.0\;\mathrm{ms}$. For these steps, OpenCV is used.

### 2.4. Hardware and Software Components

For the development of our refined architecture, the same PC hardware as in [2] is used. This allows us to compare the measurements to our initial development. For comparison with the prototype’s system, a GPU from the same generation as used there (GPU 2) is added. The components are listed in Table 1. Scharfenberger’s prototype uses more recent and faster hardware for the machine. The used software was updated to more recent versions. In comparison to our previous work, we switched to a direct integration of Nvidia TensorRT. The used software versions are listed in Table 2.

### 2.5. CNN Classifier

The classification CNN models were selected by Strelen to provide a suitable trade-off between classification performance and inference latency. A “small” and a “big” CNN were chosen, the architectures of which we are not allowed to disclose. The model input tensor has dimensions [N,64,64,4] with uint8 data type, where N∈{1,…,32} denotes the batch size. The “small” model for our inference latency tests has 0.73 M parameters and an ONNX file size of 2.9 MB. The “big” model for our inference latency tests has 1.25 M parameters and an ONNX file size of 5.2 MB.

To evaluate classification performance, the same CNN architectures were trained on a representative industrial dataset of “Müller Thurgau” berries. The dataset consists of 2450 RGBA images with a resolution of 64 px by 64 px. The images were manually selected and balanced to obtain equal class frequencies. A training/validation/test split of 70%/15%/15% was used. The dataset and CNN training results were provided by Theis [15].

The classification task comprises seven classes:

1. Good,
2. Bad,
3. Background,
4. Splash,
5. Interior,
6. Stem,
7. Medium.

For practical machine operation, the seven output classes are aggregated into a binary keep/reject decision.

The class “keep” comprises:

- Good,
- Background,
- Splash,
- Interior,
- Medium.

The class “reject” comprises:

- Bad,
- Stem.

## 3. Results

**Figure 5:**
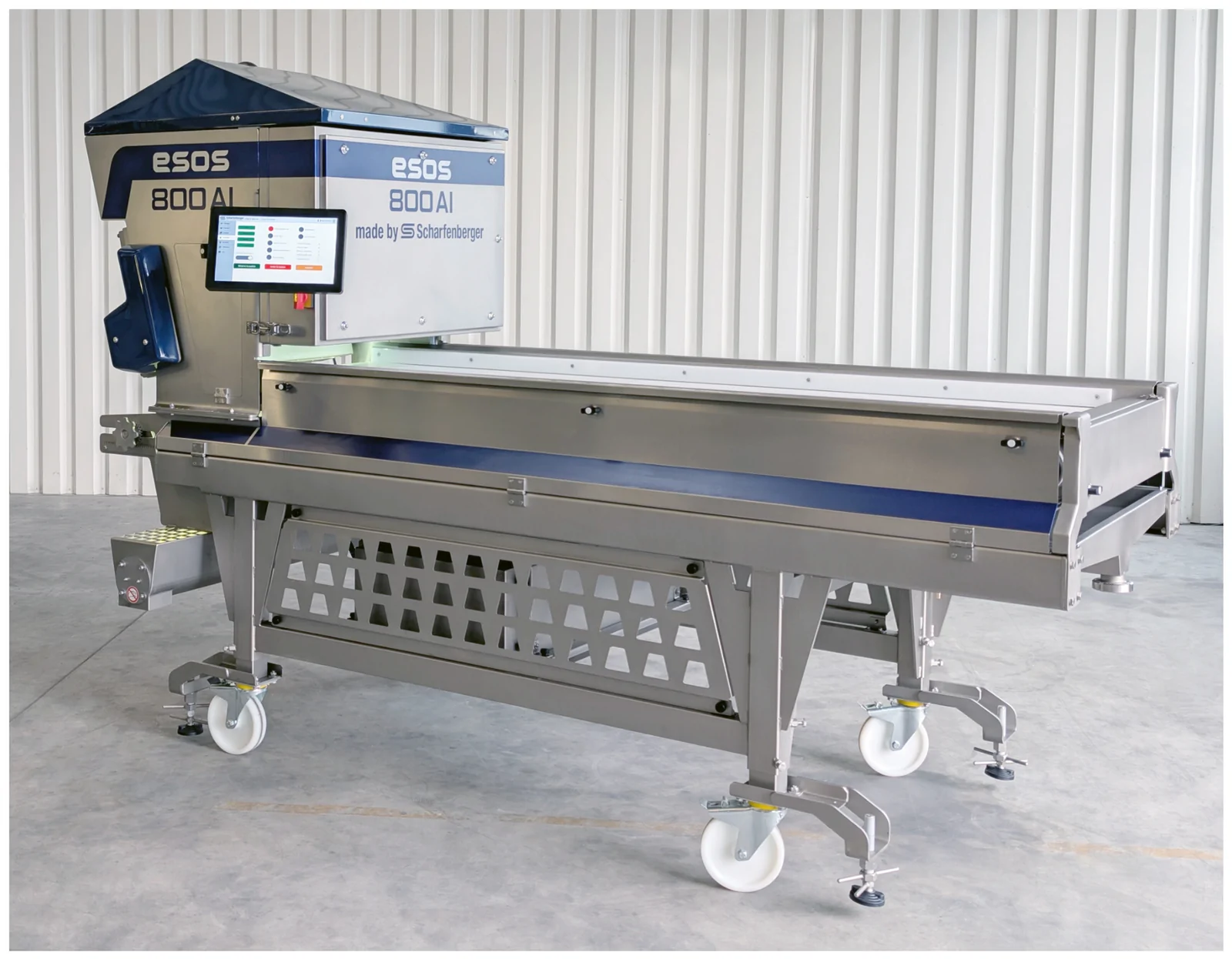
The prototype machine.

In empirical tests with Scharfenberger’s machine prototype, shown in Figure 5, a reject timing delay ($t_{max}^{\prime }$) of 13.5 ms was selected. This sets the real-time barrier for our system. There is a difference of 6.5 ms compared to our theoretical value $t_{max}=20\;\mathrm{ms}$. This difference is assumed to originate from additional delays of the reject system, including communication delays, valve response time, pneumatic transport through the tubing and nozzle assembly, and propagation of the air jet.

The camera was set to a line rate ($f_{L}^{\prime }$) of 11.5 kHz, which differs slightly from the theoretical design value of 12 kHz. This adjustment was made empirically to maintain approximately square pixels. The theoretical system latency boundary changes as follows, with $s_{p}^{\prime }$ being 128 px due to the accumulation of twice $s_{p}$ to prevent cutting objects.

(4) $$t_{max,obj}^{\prime }=t_{max}^{\prime }-s_{p}^{\prime }\cdot f_{L}^{\prime -1}=2.4\;\mathrm{ms},$$

assuming the actual belt speed $v_{b}$ is exactly the design value. $t_{max,obj}^{\prime }$ determines the time budget for the processing steps 4–12 in Figure 2.

From [11] and [2] we know that the main worst-case latency contributors are:

1. 2D Representation (3 in Figure 2), which is already accounted for in $t_{max,obj}^{\prime }$,
2. CNN Inference (9 in Figure 2),
3. Transfers from/to host (7, 11 in Figure 2).

The following measurements and assumptions are carried out to check if these constraints are met.

### 3.1. CNN Evaluation

**Figure 6:**
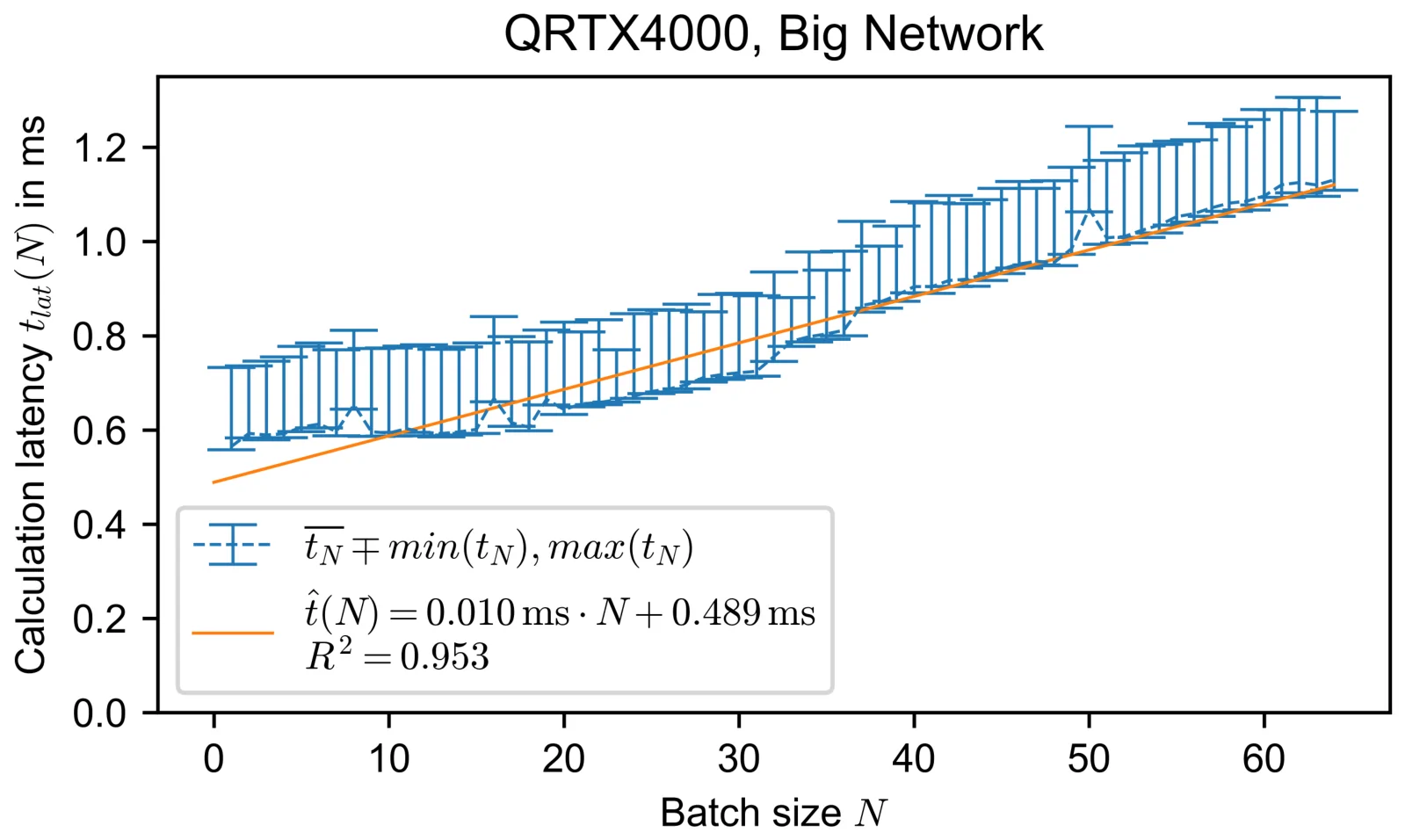
GPU1 inference time scaling with batch size. Every batch size was tested 300 000 times, the whiskers show the min-to-max range.

To assess (2), we tested the inference speed on our GPU and a GPU matching the prototype’s architecture. For this, a sweep of batch sizes from 1 to 64 was processed. Table 3 shows the timing results, and Figure 6 shows a plot for GPU1 and the “big” model. Inference time is not proportional to the batch size. A linear approximation of the measurements on GPU1 yields a slope of 10 μs per additional sample. There are batch sizes with exceptionally high inference time, which might originate from TensorRT’s optimizer. Comparing batch size 64 of “small”, GPU 2 is 1.4 times faster than GPU 1. In comparison, the speed advantage for the “big” model is only 1.14. Because the image transfer quantities are identical, the reason for the observed difference cannot be determined from the present measurements. The difference may originate from better software optimizations for “small” or the better memory throughput of GPU 1 (416.0 GB/s vs. 256.0 GB/s) or the PCIe-Bandwidth (3.0 × 16 vs. 3.0 × 8 with our CPU) compensates for the lower raw processing power (7.12 TFLOPS vs. 12.00 TFLOPS for float32) [16,17]. By adding six sigma to GPU 2’s worst latencies, we obtain 1 ms for CNN inference, leaving a 1.4 ms margin.

**Figure 7:**
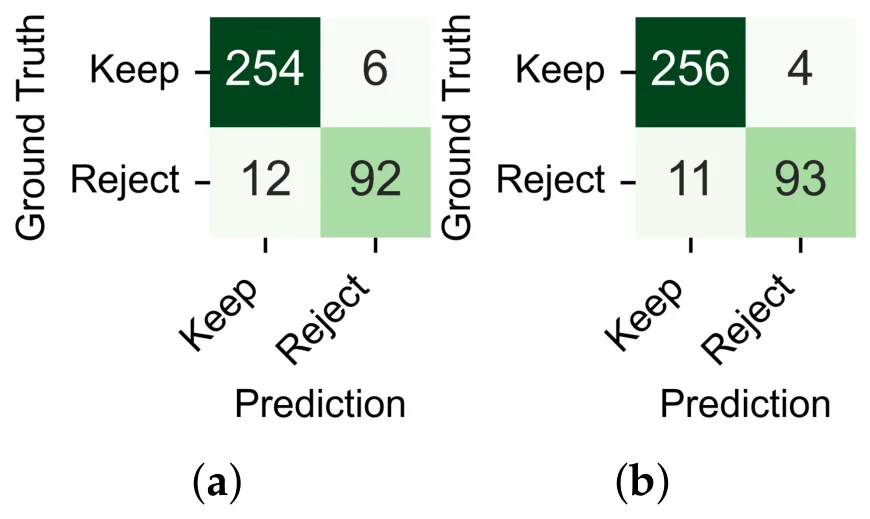
Confusion matrices for the binary keep/reject decision: (**a**) “Small” CNN. (**b**) “Big” CNN.

**Figure 8:**
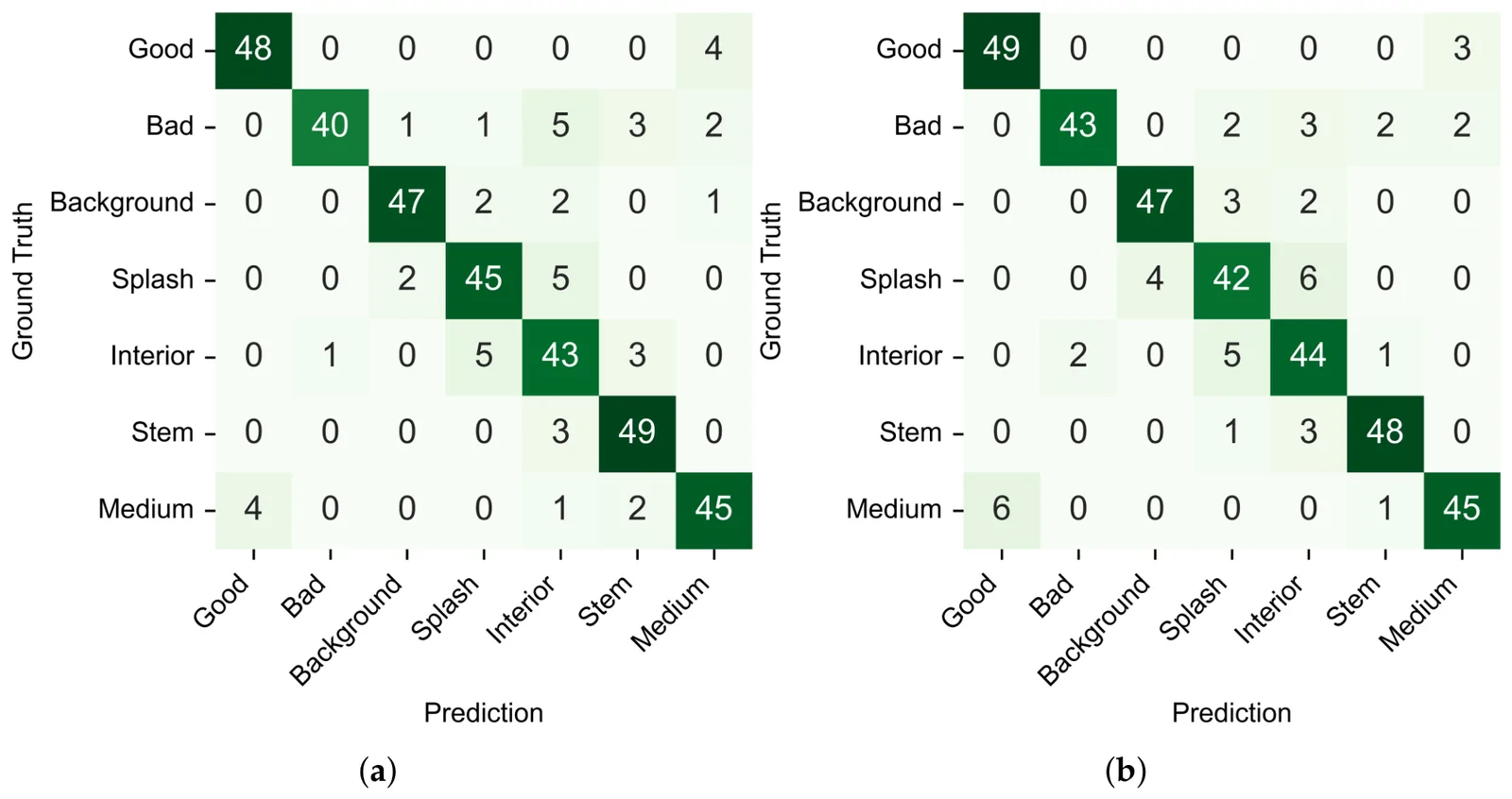
Confusion matrices for the classification task: (**a**) “Small” CNN. (**b**) “Big” CNN.

To complement the latency evaluation, the classification performance of the employed CNN architectures was evaluated on a representative industrial dataset provided by T. Theis [15]. The resulting binary keep/reject confusion matrices are shown in Figure 7, while Figure 8 shows the corresponding seven-class confusion matrices. Table 4 summarizes the binary keep/reject performance metrics.

### 3.2. FPGA Evaluation

To assess the processing latencies of our system, an FPGA design with a synthetic image source was developed. We use a real image from the application and replay it at the target line rate. The image consists of 135 objects and has 1000 lines, which results in 1552.5 objects per second or 5,589,000 per hour. The selected test image therefore represents a throughput of approximately 5.6 t/h and does not fully utilize the machine’s nominal throughput capability of up to 10 t/h.

The latency is measured between the beginning of an object in the line scan data stream and the received answer from the classifier. We used GPU1 and the big model. The resulting values are shown in Table 5. The measured maximum latencies of the laboratory setup are higher than the desired 13.5 ms. The main latency contributors are the stages from preprocessing to object extraction, stages 2 to 5 in Figure 2. While testing the prototype, there were no real-time violations. This was validated by checking an FPGA-internal counter of real-time misses. The latency breakdown shows that the host-side processing contributes only a small fraction of the total latency. However, the observed violations in the laboratory setup are also comparatively small. Therefore, a moderate reduction of the PC-side latency appears sufficient to move the system into a stable operating regime, which is consistent with the behavior observed on the prototype system. The observed performance difference between the laboratory system and the customer system may originate from factors such as the customer system’s faster hardware platform and its non-NUMA memory architecture. However, this explanation remains speculative and requires further investigation.

To overcome these latency issues, which are mainly dominated by the creation of the 2D representation within the FPGA, a new FPGA design was developed. Instead of adding two 64-line blocks together, we add three blocks of 32 lines together and repeat each 32 line block twice. This triples the data rate needed to process internally, but reduces the theoretical worst-case latency for this step to $96\;\mathrm{lines}\cdot {\left(11.5\;\mathrm{kHz}\right)}^{-1}=8.35\;\mathrm{ms}$.

The resulting values are shown in Table 6. The latency constraints can be met, and for the customer’s system, the latency margin is expected to increase. Notably, the PC-pipeline’s latencies stay roughly the same as in Table 5 even though the batch frequency increased by 33 %.

### 3.3. CPU Evaluation

To assess the CPU-based pipeline, the same image was used. The FPGA is used to emulate the line scan camera and the tracking and reject control. Stages 2 to 11 of Figure 2 are executed on the PC host. Data blocks of 16 lines are fed with a period of 1.4 ms. The result of the HSI thresholding is bit-exact to the FPGA simulation; the result after the morphological operations differs slightly. Our test image results in 140 objects, leading to 1610 objects per second. The object count difference originates from the implementation of blob analysis. While the FPGA implementation only analyzes 128 or 96 lines, the CPU’s BLOB information is retained until the end of the blob is detected. This leads to larger bounding boxes.

**Figure 9:**
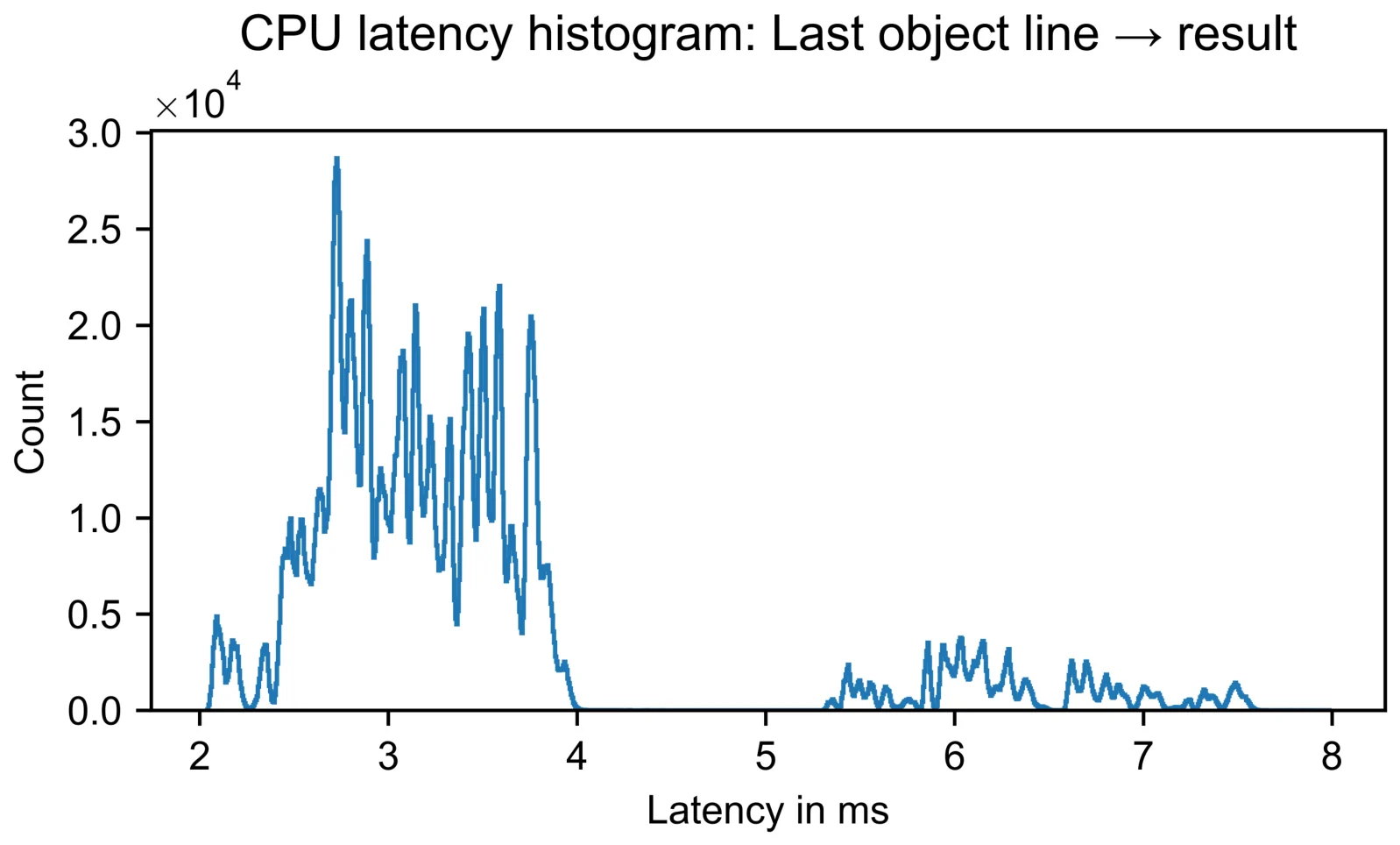
CPU implementation histogram of the latency between the last acquired object line in the FPGA and the reception of the classification result in the FPGA (“From end” measurement in Table 7).

The resulting values are shown in Table 7. The maximum measured value is 13.5 ms, which is on par with our real-time barrier. To isolate the host-side processing contribution, Figure 9 shows a histogram of the latencies from the object’s last acquired line to receiving the result in the FPGA, whose corresponding statistical values are “From end” in Table 7. The majority of values are between 2 ms and 4 ms, with another cluster between 5.4 ms and 7.5 ms and some single outliers up to 8 ms. The bimodal distribution and the observed outliers indicate that additional latency sources are present in the host system. Potential causes include NUMA effects or memory bandwidth limitations, which require further investigation. Even though all observed latencies remained below or matched the real-time boundary, this solution cannot guarantee a fail-safe response to real-time violations, unlike the FPGA implementation. This leaves a reliability gap, as deterministic timing guarantees cannot be established in the same way as for the FPGA-based implementation, even if the observed latency outliers are eliminated.

## 4. Discussion

We showed how our system concept translates into the practical application of grape sorting. In the project, three parties worked together to achieve a user-configurable CNN-based sorting machine. As expected, the main latency contributor is the formation of the 2D representation. The maximum latency observed with our lab system of 14.0 ms is higher than the real-time limit of 13.5 ms. However, while working with the prototype, we did not encounter real-time violations, suggesting that the faster hardware used in the prototype is below the barrier. We expect only a short margin to the real-time barrier. To accommodate this, a change in the FPGA design was implemented and evaluated, which lowers the measured worst-case latency to 11.3 ms. According to [18], the prototype’s material throughput reached 8–10 metric tons per hour. In qualitative field tests performed together with selected winegrowers, the prototype showed the expected throughput and qualitative sorting performance. Shifting the FPGA image processing pipeline to the host CPU proved feasible when using highly optimized processing algorithms. However, at the current line rate, it is operating at the limits of what is possible on our test system. The FPGA solution was successfully tested with higher line frequencies and throughput in [2]. Besides the achievable throughput, the FPGA architecture provides deterministic timing and low-jitter reject control, making it particularly attractive for real-time sorting applications.

The distance between the line scan camera and nozzle array will be adjusted, or the system’s latency will be reduced, to gain a larger timing margin. Future work will focus on a quantitative evaluation of sorting performance and the investigation of additional spectral bands such as UV, NIR, or SWIR. This may help if some of the information is not embedded in the RGB band. Previous work demonstrated that information related to grape maturity and sugar concentration is present in VIS, NIR, and SWIR spectral bands [19,20]. In [21], UV-induced fluorescence was investigated for the detection of grey mold (Botrytis cinerea) in grape berries.

The demonstrated CPU-based image processing pipeline suggests that lower-cost FPGA-free systems may be feasible at line rates of up to 12 kHz, particularly when using a UMA architecture. Camera interfacing, tracking, and reject control were not part of this evaluation and require further investigation.

Addressing the research questions posed in this work, we conclude:

1. The hybrid image processing architecture is suitable for grape sorting applications. The demonstrated system satisfies the real-time requirements while providing the flexibility of a user-trainable classification CNN.
2. Our test system achieved a worst-case latency of 14.0 ms. Despite exceeding the defined real-time barrier, the system operated without observed real-time violations on the customer’s hardware and achieved an estimated throughput of 8–10 t/h.
3. Host-side preprocessing proved feasible at a line rate of 11.5 kHz. This suggests that lower-cost FPGA-free architectures may be possible for moderate throughput applications, but at the cost of losing hard real-time capability and the need to implement object tracking elsewhere.
4. An improved FPGA architecture reduced the measured worst-case latency from 14.0 ms to 11.3 ms on our test system. This potentially provides an additional timing budget for cheaper hardware or bigger CNNs.

## Figures and Tables

**Table 1:** System hardware components.

Component	Model/Configuration
Frame grabber	Basler imaFlex CXP-12 Quad
GPU 1	Nvidia Quadro RTX 4000
GPU 2	Nvidia RTX 2000 Ada Generation
CPU	AMD EPYC 7551P
Mainboard	Supermicro H11SSL-I Rev. 2.0
RAM	8 channels of 8 GiB DDR4-ECC-Reg 2133 MHz

**Table 2:** System software components.

Component	Model/Configuration
Operating system	Debian 13 with PREEMPT_RT 6.12.57
GPU driver	Nvidia 575.51.03
CUDA	12.9
TensorRT	10.14.1.48

**Table 3:** CNN processing timings measured in ms. Prefix 1 denotes GPU 1, prefix 2 denotes GPU 2. Every batch size was tested 300,000 times. The ± value denotes the standard deviation.

Batch Size	1: Small	1: Big	2: Small	2: Big
1	0.213 ± 0.002	0.565 ± 0.004	0.190 ± 0.002	0.528 ± 0.002
2	0.301 ± 0.002	0.593 ± 0.004	0.183 ± 0.002	0.557 ± 0.002
32	0.567 ± 0.003	0.756 ± 0.004	0.391 ± 0.002	0.650 ± 0.002
64	0.912 ± 0.005	1.131 ± 0.006	0.638 ± 0.002	0.990 ± 0.002

**Table 4:** Binary keep/reject classification performance of the evaluated CNN models. Accuracy, Precision, Recall, and F1-score are computed from the aggregated keep/reject confusion matrices shown in Figure 7. Values are given in percent.

	Small	Big
Accuracy	95.1	95.9
Precision	93.9	95.9
Recall	88.5	89.4
F1-score	91.1	92.5

**Table 5:** FPGA implementation timings measured in ms. Every measurement sample count is 4,013,664. “Complete” denotes the latency between object appearance in the line scan stream and the class prediction from the PC is received (Stage 2–12 in Figure 2). “From end” denotes the same latency as “Complete”, but from the last line of the object. “Start-Readout” denotes the latency between object appearance in the line scan stream and when it is extracted as an object patch for classification (Stage 3–5 in Figure 2). “End-Readout” denotes the same latency as “Start-Readout”, but from the last line of the object. “PC-Pipeline” is the latency measurement between patch extraction and receiving the answer from the PC (Stage 5–12 in Figure 2).

Measurement	Mean	Min	Max	Median	99.9%-Perc
Complete	10.7	4.0	14.0	11.1	13.2
From end	4.2	1.5	8.6	4.0	7.6
Start-Readout	9.5	2.9	11.9	9.9	11.9
End-Readout	2.9	0.7	6.3	2.8	6.3
PC-Pipeline	1.2	0.8	2.3	1.2	1.6

**Table 6:** Improved FPGA implementation timings measured in ms. Every measurement’s sample count is 4,675,500. “Complete” denotes the latency between object appearance in the line scan stream and the class prediction from the PC is received (Stage 2–12 in Figure 2). “From end” denotes the same latency as “Complete”, but from the last line of the object. “Start-Readout” denotes the latency between object appearance in the line scan stream and when it is extracted as an object patch for classification (Stage 3–5 in Figure 2). “End-Readout” denotes the same latency as “Start-Readout”, but from the last line of the object. “PC-Pipeline” is the latency measurement between patch extraction and receiving the answer from the PC (Stage 5–12 in Figure 2).

Measurement	Mean	Min	Max	Median	99.9%-Perc
Complete	9.0	3.9	11.3	9.8	10.6
From end	2.8	1.5	5.7	2.6	4.7
Start-Readout	7.8	2.8	9.0	8.8	9.0
End-Readout	1.6	0.7	3.4	1.4	3.4
PC-Pipeline	1.1	0.7	2.3	1.2	1.9

**Table 7:** PC host implementation timings measured in ms. “Complete” denotes the latency between object appearance in the line scan stream and the class prediction from the PC is received (Stage 2–12 in Figure 2). “From end” denotes the same latency as “Complete”, but from the last line of the object. “Pre-Extract” is the latency measurement between 16-line input and object batch output (Stage 2–5 in Figure 2).

Measurement	Mean	Min	Max	Median	99.9%-Perc	Count
Complete	7.3	4.0	13.5	7.5	13.0	3,586,985
From end	3.4	2.0	8.0	3.2	7.5	3,586,985
Pre-Extract	1.1	1.0	1.4	1.1	1.2	300,000

## Data Availability

The datasets generated and analyzed during the current study are not publicly available due to confidentiality restrictions. Representative measurement data derived from non-confidential example datasets may be made available by the corresponding author upon reasonable request.
